# Unique RNA Gene Expression Profile Is Seen in Chronic Non-Specific Low Back Pain

**DOI:** 10.3390/ijms27010287

**Published:** 2025-12-27

**Authors:** Ann-Christin Sannes, Imran Amjad, Jenna Duehr, Usman Ghani, David Rice, Heidi Haavik, Imran Khan Niazi, Torgeir Moberget, Johannes Gjerstad

**Affiliations:** 1 Faculty of Health Science, Oslo Metropolitan University, 0176 Oslo, Norway; 2 Mental Health Services, Akershus University Hospital, 1478 Nordbyhagen, Norway; 3 Faculty of Rehabilitation and Allied Health Sciences, Riphah International University, Islamabad 44000, Pakistan; 4 Center for Chiropractic Research, New Zealand College of Chiropractic, Auckland 1060, New Zealand; 5 Department of Exercise Science, Faculty of Science, University of Auckland, Auckland 1010, New Zealand; 6 Faculty of Health and Environmental Sciences, Auckland University of Technology, Auckland 1010, New Zealand; 7 Waitemata Pain Services, Te Whatu Ora—Health New Zealand Waitematā, Auckland 0622, New Zealand; 8 Health and Rehabilitation Research Institute, Auckland University of Technology, Auckland 1010, New Zealand; 9 Center for Sensory-Motor Interaction, Department of Health Science and Technology, Aalborg University, 9220 Aalborg, Denmark; 10 Centre for Precision Psychiatry, University of Oslo, 0316 Oslo, Norway; 11 Faculty of Health Science, Kristiania University, 0107 Oslo, Norway

**Keywords:** low back pain, RNA sequencing, sensitization, inflammation

## Abstract

Previous reports suggest that the progression from subacute to chronic non-specific low back pain (nsLBP) involves functional changes in both the nervous and immune systems. The purpose of the present study was to characterize the gene expression profiles of circulating immune cells that affect the interaction between these two systems when subacute nsLBP turns into chronic nsLBP. Participants aged 18–55 were included based on the presence or duration of LBP, with peripheral blood mononuclear cells collected for RNA sequencing from 20 healthy controls (no nsLBP), 20 subclinical patients (intermittent nsLBP), and 19 chronic patients (long-term nsLBP). The data revealed that chronic nsLBP is linked to a distinct gene expression profile, with 139 uniquely differentially expressed genes (DEGs), differing from those in the subclinical and control groups. Interestingly, comparing chronic and subclinical groups showed minimal overlap in DEGs, indicating a clear inflammatory distinction between subclinical nsLBP and chronic nsLBP. The findings also indicated that patients with chronic nsLBP were different from other individuals regarding axon guidance, indicating neuroplastic changes when intermittent nsLBP turns into chronic nsLBP. Hence, early recognition of the transition from subclinical to chronic nsLBP using RNA profiling may pave the way for more precise therapeutic strategies targeting neuroplastic changes and inflammatory processes.

## 1. Introduction

Previous studies show that about 90% of LBP cases are classified as non-specific LBP (nsLBP) cases, where the pain is of unknown origin [1]. For some patients, nsLBP is recurrent in nature and characterized by periods of intermittent pain separated by periods of recovery with no symptoms [2]. Such intermittent nsLBP is often referred to as subclinical pain [3]. Unfortunately, the subclinical subset of nsLBP cases may progress to persistent cases, also called chronic nsLBP [4,5,6]. To prevent this, it is essential to better understand the inflammatory and neuronal processes [7,8] involved in the development of such chronic LPB.

Although it is believed that the transition from subclinical to chronic nsLBP may involve long-term changes in the nervous system [7,9], the mechanisms underlying the development of chronic nsLBP are still unclear. Hence, there is a need to further investigate potential molecular differences that might assist in explaining the pathophysiology of nsLBP. Moreover, nsLBP is often accompanied by co-occurring musculoskeletal pain [10,11], which impacts the overall prognosis [12]. Therefore, to uncover the impact of nsLBP alone, uncovering and excluding those with co-occurring musculoskeletal pain is of importance.

The development and maintenance of chronic nsLBP might involve an interplay between the nervous system and the immune system [13]. These two systems speak a common biochemical language, involving shared ligands such as neuroendocrine hormones and many cytokines. The shared signaling molecules act on both sensory neurons and immune cells, influencing regulation of pain at the molecular level. Moreover, previous data show that peripheral efferent nerve fibers that innervate lymphoid organs (e.g., the spleen) with noradrenergic and acetylcholinergic signaling affect circulating immune cells [14,15].

Interestingly, immune cells such as leukocytes produce neuropeptides that may both suppress (e.g., endorphins and enkephalins) and facilitate (e.g., substance P) nociceptive processes [16]. In addition, evidence exists that activation of sensory nociceptors in lymph nodes may alter the transcriptome of innate immune cells [17]. This demonstrates a complete neuroimmune circuit. Hence, the immune system is an important part in the regulation of pain, which includes both development and resolution of pain [15].

Despite previously uncovered genetic markers for LBP through genome-wide association studies [18], the use of RNA seq technology in investigating chronic nsLBP has previously only been conducted using whole blood [19,20]. Therefore, the differential gene expression in the immune cells of patients with chronic non-specific low back pain (nsLBP) compared to those without pain warrants further investigation [19,21]. The aim of the present study was to examine the difference in gene expression profile in peripheral blood mononuclear cells (PBMC) between healthy controls, subclinical nsLBP, and chronic nsLBP individuals.

## 2. Results

### 2.1. Participant Inclusion Characteristics

**Figure 1:**
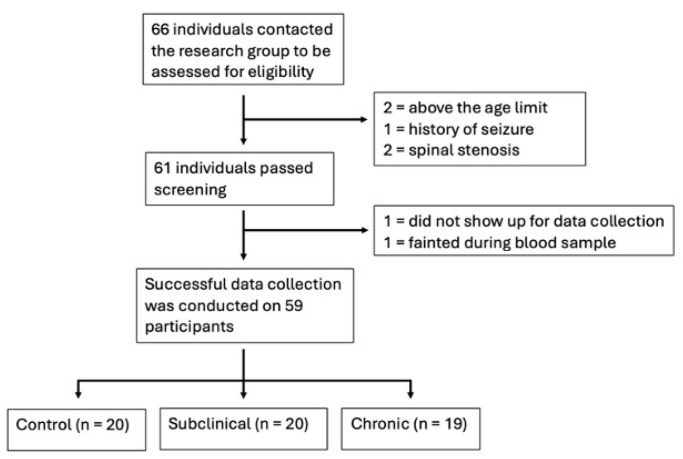
Flowchart of inclusion of participants.

A total of 59 participants (see Figure 1) were included in the study, of which 28 (47%) were women, and 31 (53%) were men, with an average age of 32.1 (SD 9.06). The three groups consisted of 20 controls, 20 subclinical, and 19 with chronic nsLBP (see Table 1). Upon quality control of mRNA isolation, all samples were deemed of sufficient quality; hence, all 59 samples were included in the analyses. Group comparisons of baseline characteristics can be seen in Supplementary Materials Table S1.

### 2.2. Gene Expression Analyses’ Results

**Figure 2:**
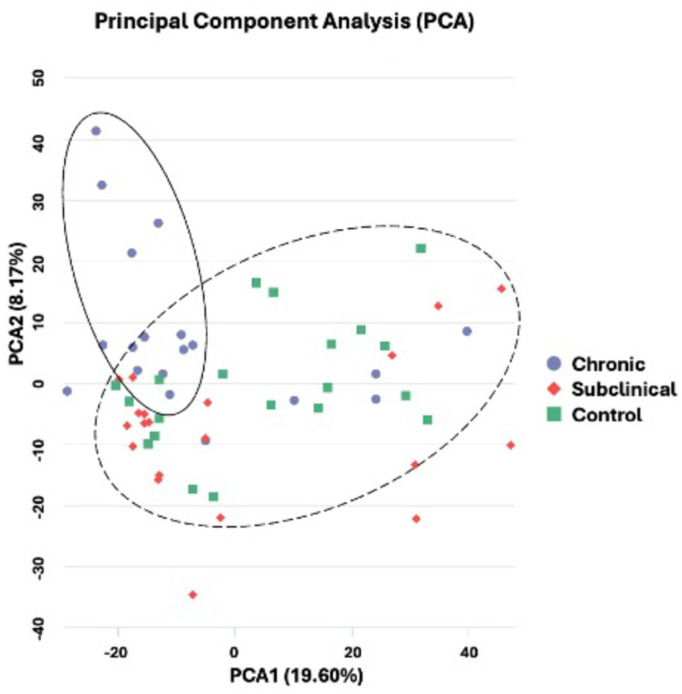
Principal component analyses (PCA) of all three groups, solid circle indicates the cluster of the chronic group, dashed circle indicates the cluster of the other two groups.

**Figure 3:**
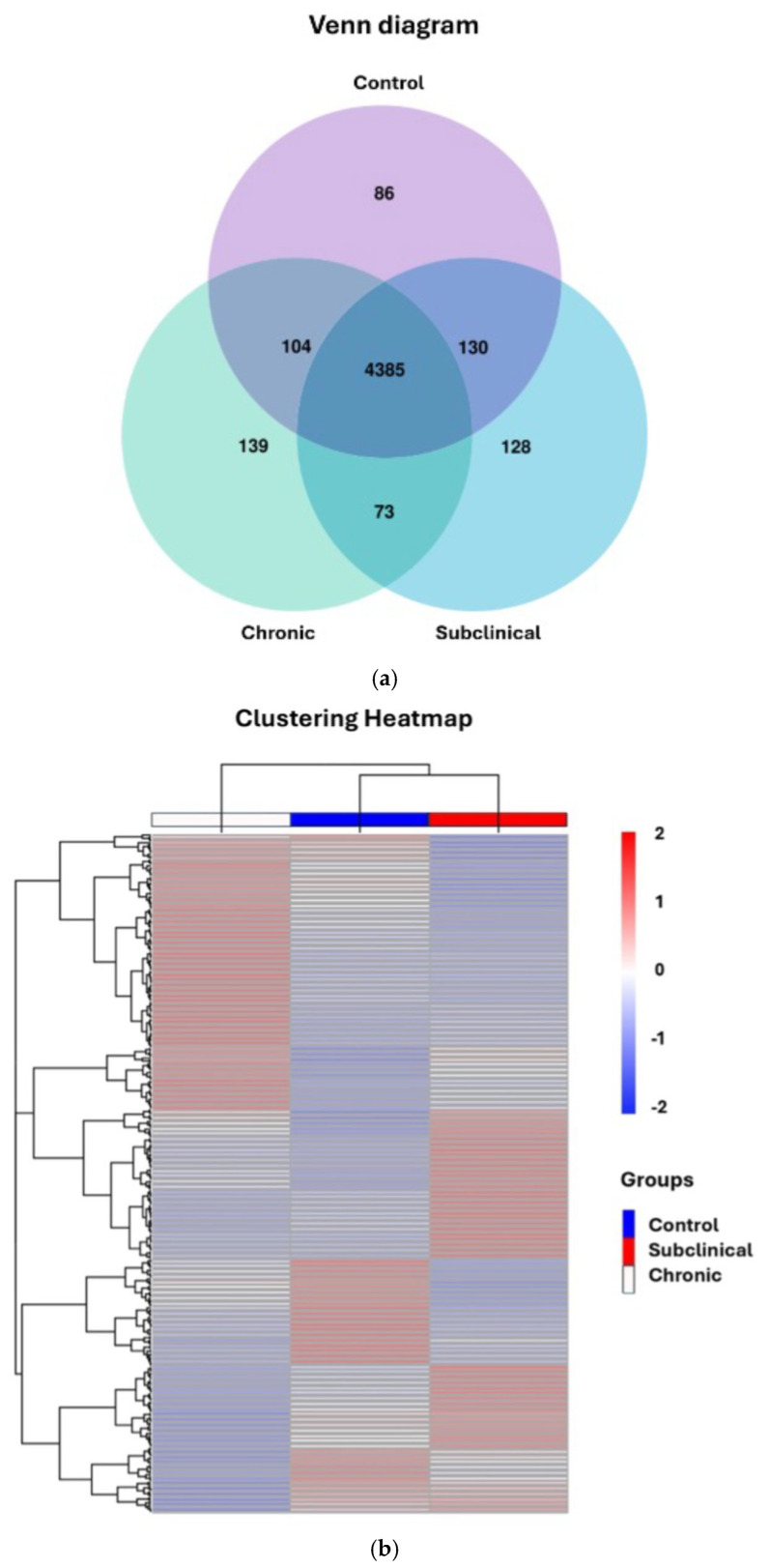
(**a**) Venn diagram showing uniquely expressed genes for all three groups: 86, 128, and 139 genes. (**b**) Heatmap of uniquely expressed genes between the three groups (n = 353).

All samples were included in a principal component analysis (PCA), indicating a clustering of participants with chronic nsLBP (see Figure 2). Utilizing a Venn diagram analysis, we identified 86, 128, and 139 unique genes expressed exclusively in the control (n = 20), subclinical (n = 20), and chronic group (n = 19), respectively. Notably, only 73 genes were co-expressed in both the subclinical and chronic groups (Figure 3a). An overview of the uniquely expressed genes for each group can be seen in Supplementary Materials Table S2. A hierarchical clustering heatmap was created using the uniquely expressed genes uncovered in the Venn diagram, showing a clustering between the control and the subclinical group (Figure 3b).

**Figure 4:**
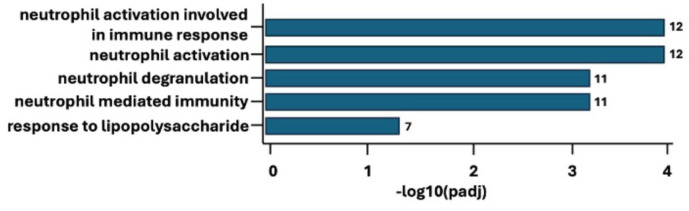
Gene ontology (GO) enrichment analyses for the chronic group—biological processes. padj—*p*-adjusted. Log2Foldchange = 1. padj < 0.05.

Next gene ontology (GO) enrichment analyses were conducted on each of the three groups separately, i.e., each group versus a reference (all samples). The analyses were performed without direction of pathway regulation (i.e., if there is an up- or down-regulation), but they provide a representation of deregulated enrichments. For the chronic group (see Figure 4), the findings were mainly associated with biological process (BP). The top three significant findings pertaining to BP enrichment in the chronic group were neutrophil activation involved in immune response (padj = 0.00012), neutrophil activation (padj = 0.00012), and neutrophil degranulation (padj = 0.00051). In contrast, for the subclinical group, no significant enrichment was found (see Supplementary Materials Figure S2 and Table S4). This indicates only small differences regarding gene expression between the subclinical and control groups. Finally, for the control group, the top three findings were mitochondrial gene expression (padj = 0.002), mitochondrial translation (padj = 0.020), and mitochondrial RNA metabolic process (padj = 0.043). As these deregulated pathways (see Supplementary Materials Figure S1 and Table S3) highlight the contrast between the healthy subjects and the pain patients, their RNA profile represents normality or is a marker of good health.

### 2.3. Gene Expression Analyses’ Results on Only nsLBP

**Figure 5:**
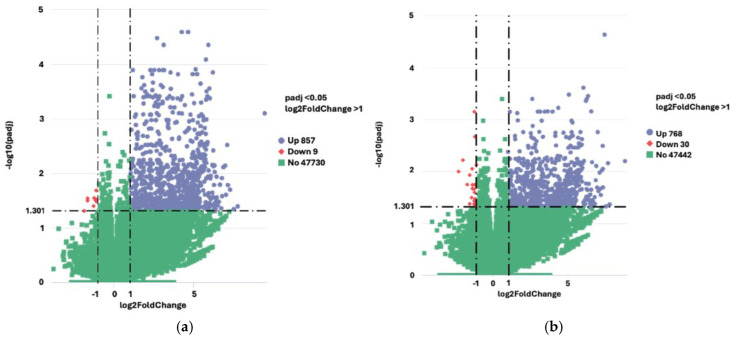
(**a**) Vulcano plot of differentially expressed genes (DEGs) comparing participants with only chronic low back pain to controls. (**b**) Vulcano plot of differentially expressed genes (DEGs) comparing participants with only chronic low back pain to subclinical low back pain.

Additional gene ontology (GO) enrichment analyses were conducted on only a subset of subjects, that is, those who reported only nsLBP (n = 10) versus a specific relevant reference, i.e., first the control group (n = 18), and next the subclinical group (n = 11). The analyses showed that the DEG gene count for the contrast chronic versus control was 9191, whereas the DEG gene count for the contrast chronic versus subclinical group was 10,570. Of these, when comparing the chronic group to the control, and the chronic group to the subclinical group, 866 and 798 significant DEGs were uncovered, respectively (see Figure 5a,b). No significant DEGs were seen when comparing the subclinical group to the controls (Supplementary Materials Figure S3).

**Figure 6:**
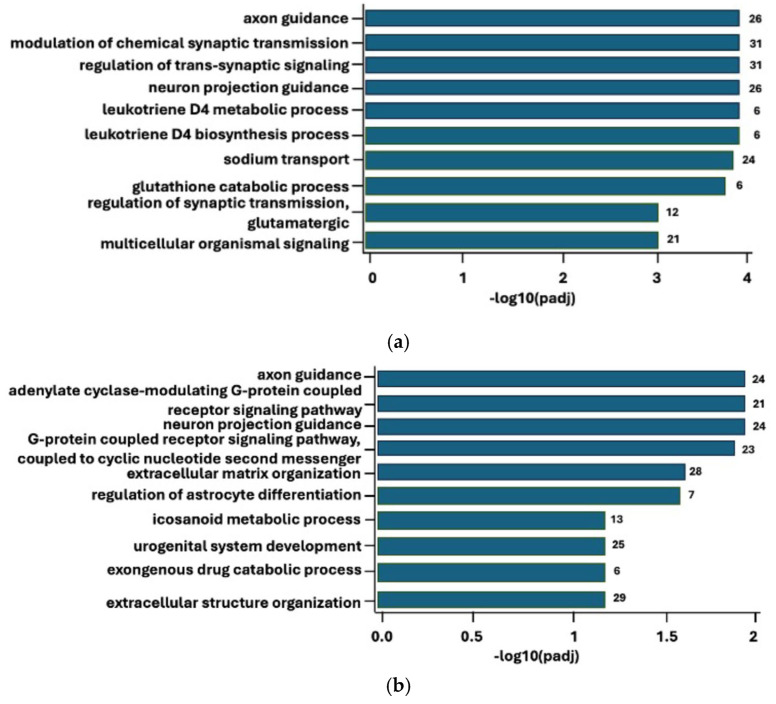
(**a**) Gene ontology (GO) enrichment analyses comparing participants with only chronic low back pain to control—biological processes. padj—*p*-adjusted. Log2Foldchange => 1. padj < 0.05. (**b**) Gene ontology (GO) enrichment analyses comparing participants with only chronic low back pain to subclinical low back pain—biological processes. padj—*p*-adjusted. Log2Foldchange => 1. padj < 0.05.

Moreover, an assessment of the most significant biological processes was conducted using a gene ontology (GO) enrichment analysis (see Figure 6a,b). Upon comparing the chronic to the control group, the top four most statistically significant biological processes were axon guidance, regulation of trans-synaptic signaling, modulation of chemical synaptic transmission and neuron projection guidance (Table 2, top). The chronic group compared to the subclinical group showed axon guidance, adenylate cyclase-modulating G-protein-coupled receptor signaling pathway, neuron projection guidance, and G-protein-coupled receptor signaling pathway coupled to cyclic nucleotide second messenger as the top four statistically significant biological processes (Table 2, bottom). Due to the lack of statistically significant DEGs, no comparison could be made between the subclinical and the control group.

### 2.4. Subsequent KEGG Pathway Analysis

Subsequent Kyoto Encyclopedia of Genes and Genomes KEGG pathway analysis of the deregulated genes for the entire sample’s chronic group (n = 19) compared to the control group (n = 20) presented 20 statistically significant pathways (Supplementary Materials Table S5) of which the top five were protein digestion and absorption (padj = 7.57 × 10^−6^), neuroactive ligand–receptor interaction (padj = 1.05 × 10^−5^), cholinergic synapse (padj = 0.0012), glutamatergic synapse (padj = 0.0013), and axon guidance (padj = 0.0031) (Table 3). KEGG pathway analysis comparing the chronic to the subclinical group that only reported nsLBP only showed four statistically significant pathways: neuroactive ligand–receptor interaction (padj = 0.0004), notch signaling pathway (padj = 0.0140), protein digestion and absorption (padj = 0.0140) and ABC transporters (padj = 0.0484) (see Table 3). Due to the lack of statistically significant DEGs, no comparison could be made between the subclinical (n = 20) and the control group.

## 3. Discussion

The present study demonstrated that chronic nsLBP was associated with a distinct gene expression profile, i.e., 139 uniquely differentially expressed genes (DEGs), different from that seen in the subclinical or the control group. Moreover, the comparison between the chronic and subclinical group showed a limited overlap of the DEGs, suggesting clear molecular differences between those with intermittent versus chronic symptoms. Hence, our findings show that the RNA profile associated with chronic nsLBP may differ from the RNA profile seen in those with intermittent nsLBP problems. Additionally, in individuals who reported only nsLBP, the chronic group was associated with the activation of the axon guidance pathway, which has been previously linked to nerve growth changes and neuroplasticity. Thus, the present data suggest that chronic nsLBP may be associated with molecular signaling pathways related to neuroplasticity.

The initial GO analysis showed that the most significant biological processes activated in the chronic group all pertained to neutrophil function and activity. This unique molecular RNA profile indicates an altered immune activity in this group. Thus, the present observations suggest a different biological trajectory than was seen in the subclinical group and/or healthy controls. GO enrichment analysis did not present any significant findings for the subclinical or the control group, despite uncovering 128 and 86 DEGs, respectively. This lack of significant pathway enrichment and unsupervised clustering indicates that the subclinical group and the control group may share a more closely related expression pattern than what was observed in the chronic group. However, the extent to which these characteristics observed in peripheral immune cell gene expression reflect neuroplastic changes in the brain associated with chronic nsLBP remains to be investigated.

Importantly, circulating neutrophils have been characterized as pronociceptive [22] and are known mediators of nociceptor sensitization through release of proinflammatory cytokines [23]. However, neutrophils may also have an anti-nociceptive role, and their up-regulation in the early phase of an acute LBP episode appears critical to LBP resolution [20]. Although the mechanism behind this link is still not fully understood, our findings align with previous evidence indicating a known link between the immune system and chronic pain [20,22,24] and suggest that neutrophil regulation may play an important role maintaining pain, likely through the ongoing sensitization of nociceptive pathways and/or through maladaptive central plastic changes.

Further, the PNS has been found to play a direct and active role in modulating innate and adaptive immunity [25]. Previous findings propose that pro-inflammatory cytokines released by monocytes can affect the brain in different ways. This could be through leaky regions of the blood–brain barrier (humoral pathway), by stimulating afferent nerve fibers in the vagus nerve (neural pathway), and by stimulating microglia that recruits monocytes into the brain (cellular pathway) [26,27]. Furthermore, the immune system has repeatedly been associated with the induction and resolution of pain via interactions with the nervous system [28,29,30]. Importantly, neuro-immune alterations in the PNS and the CNS play a role in the pathophysiology of chronic pain [31].

Moreover, the experience of pain can persist long after the initial cause of injury has healed, representing an adapted dysfunction, e.g., sensitization, of nociceptive pathways in the PNS and/or the CNS [7,23]. Several molecular and biophysical mechanisms contribute to the phenomenon of sensitization in peripheral axons [8,23]. Previous research implies that chronic pain perception is associated with phenotypic changes that are expressed at all levels (primary afferents to cortex) and alter pain modulation [7,9]. This might be especially true for those suffering with chronic nsLBP, where no apparent structural cause to explain the pain can be detected.

The presence of co-occurring musculoskeletal pain seen with LBP could influence the findings seen in the initial GO enrichment analysis in this study. Hence, to uncover a clearer picture of LBP without other types of pain and comorbidity, additional analyses were conducted on those with only nsLBP, i.e., participants who did not report any co-occurring musculoskeletal pain, leaving out those reporting pain elsewhere.

In these additional analyses, comparing the chronic group with only nsLBP to the control group, and the chronic group with the subclinical group, 866 and 798 deregulated DEGs were uncovered, respectively. The GO enrichment analysis of these DEGs presented statistically significant biological processes all relating to the nervous system. The most significant process for both analyses was again axon guidance, a critical step in the wiring of the nervous system during development [32] that is also found to be active in a mature nervous system [33,34,35]. Axon guidance cues encompass axonal and neuronal sprouting [14,20], synaptic plasticity [35], and the refinement of neural networks [35,36]. In particular, neural sprouting, which can occur in response to altered requirements by the nervous system or injury [37,38], might be an important aspect of neuroplasticity that promotes chronic pain.

To further investigate the mechanisms underlying chronicity in the chronic group with non-specific low back pain (nsLBP), subsequent KEGG pathway analyses were performed. These analyses revealed that, in the comparison between the chronic nsLBP group and the control group, the most deregulated pathways were associated with protein digestion and absorption, neuroactive ligand–receptor interaction, cholinergic and glutamatergic synapses, and axon guidance. These findings are consistent with previous data from rodent models of pain [39].

Molecules found in the axon guidance pathway have been found to modulate synaptic plasticity, important in chronic pain, and also essential for early stages of long-term potentiation (LTP) [40]. This might be an indication that this pathway may have a role in maintaining chronic pain. Moreover, three of the other significant pathways, namely the glutamatergic synapse, the cholinergic synapse, and neuroactive ligand–receptor interaction, are known to act on either pain modulation, neuroplasticity, or both. Glutamate and glutamate synapses are present in many regions of the nervous system and play a crucial role in neuronal signaling, including the sensation of pain [41,42]. Interestingly, glutamate receptors such as the N-methyl-D-aspartate (NDMA) receptor have also been found to be important in LTP [43] and chronic pain [42]. Furthermore, the cholinergic synapse [43] and the neuroactive ligand–receptor interaction [44] have also been linked to chronic pain modulation. Hence, considering that four of the five most significant pathways uncovered in this study all relate to either neuroplasticity, pain modulation, or both, it seems probable that those with chronic nsLBP present with different neurophysiological function and activity than those without chronic nsLBP.

Furthermore, upon comparing the chronic group to the subclinical group, four significant pathways emerged. Two of these overlapped with the comparison between the chronic and control group, namely protein digestion and absorption, and neuroactive ligand–receptor interaction. In addition, two other significant pathways were uncovered, notch signaling pathway and ABC transporter. The notch signaling pathway is important in several developmental processes, such as the regulation of peripheral immune cells [45,46], and plasticity in the adult brain [47]. This is in line with the two overlapping pathways found in the KEGG analyses. The last and final pathway, the ABC transporter pathway, is involved in membrane transport [48] of substrates such as lipids and proteins [49] across different cell membranes, in addition to the BBB [50]. Both acute and chronic pain have been found to alter the ABC transporter functional expression, through for example, protein tight junctions, across the BBB [50,51]. Hence, one could speculate that this pathway may represent a link to the humoral pathway mentioned above. If so, altered permeability in the BBB, as a result of, e.g., immune cells [51], could potentially influence the CNS by the altered immune activity seen in the chronic group [52]. However, potential underlying mechanisms to confirm such a theory would need further research.

### Strengths and Limitations

One of the strengths of this study is the number of participants in each group, providing more than double the number required to achieve acceptable power for the RNA seq analyses [53]. Furthermore, by isolating the PBMCs, a clearer picture can be presented of any changes that might be found in the immune system. However, with this method of isolating the PBMCs only a few certain types of white blood cells are included, i.e., lymphocytes, monocytes, and dendritic cells [54]. Hence, a more detailed picture of the gene expression in specific cell types remains to be investigated. While peripheral blood sampling offers valuable insights, more detailed information regarding potential CNS molecular changes and/or differences remains unknown.

Another limitation is the cross-sectional design that does not provide information on how these changes evolve over time, e.g., prior to turning chronic or changes over time after reaching a chronic state. However, the present paper constitutes the initial report from an ongoing perspective cohort study and will be followed up. Moreover, the average level of pain intensity was relatively low for all included participants. It is not known to what extent intensity, and not only longevity, of pain is important for gene expression differences. Additionally, we used a solely a transcriptomic approach. Examination of protein expression through proteomics has yet to be conducted. Hence, future research should attempt to include such participants to uncover nuances of influencing factors on gene expression, but also protein expression (cytokines), in LBP sufferers.

## 4. Materials and Methods

This present paper constitutes the initial report from a larger ongoing prospective cohort study, which encompasses repeated multiple assessments that will be carried out over a prolonged period. The overall study design, power calculation, participant recruitment, data collection, and sample processing were described in a recently published protocol [53].

### 4.1. Participant Recruitment

Participants were recruited through Facebook; word of mouth; and flyers distributed in private chiropractic clinics, factories, police and ambulance services, the central universities and libraries, and the spinal center and hospital in Auckland, New Zealand. The recruitment period was between April and November 2024. Participants were included if they were between 18 and 55 years old, had either no nsLBP (control group), current or recurrent low back ache, pain, or tension of low intensity that lasts less than 3 months that they have not sought treatment for (subclinical group) or moderate-to-severe chronic nsLBP, i.e., nsLBP that has lasted 3 months or longer (chronic group). Participants were excluded if they were currently experiencing seizures, cancer, psychiatric disease, pregnancy, rheumatic disease, cauda equina, spinal stenosis, or sciatica, or used medication such as sedatives, muscle relaxants, or sleep medication. Notably, subclinical patients did not experience persistent pain lasting more than 3 months, but some of them had episodes of intermittent pain, with brief pain periods occurring between 3 and >12 months ago. The study was approved by the New Zealand Health and Disabilities Ethics Committee (HDEC, reference: 2023 EXP 19096).

### 4.2. Data Collection

After signing the consent form, the participants were asked to indicate the length of LBP complaint and were thereafter assigned group belonging (controls, subclinical, or chronic). A blood sample was collected from the median cubital vein by a Registered Nurse prior to PBMC preparation and storage. Baseline characteristics were then collected through multiple questionnaires, such as gender, age, ethnicity, education level, pain catastrophizing, disability, kinesiophobia, and use of pain medication for their LBP.

### 4.3. Blood Sample Processing and Analyses

Each blood sample was collected in a 10 mL BD Vacutainer lithium heparin tube using BD Vacutainer^®^ Push Button Blood Collection (Becton, Dickinson and Company, Franklin Lakes, NJ, USA) set 21 g with Holder. Isolation of Peripheral Blood Mononuclear Cell (PBMC) was performed within 10 min after blood collection, using STEMCELL (STEMCELL Technologies, Vancouver, BC, Canada) SepMate 50 mL tubes according to their protocol. The resulting cell pellet was then resuspended prior to preparation for storage. Preparation for cryopreservation was conducted using CryoStor^®^CS10 (STEMCELL Technologies, Vancouver, BC, Canada). All samples were stored at −80 °C prior to shipping. Once all the samples were collected, they were shipped at −80 °C, using a specialized courier (Cencora, World Courier Limited, London, UK), to the commercial company Novogene Co., Ltd., in Cambridge, the UK, for mRNA isolation and mRNA seq.

Novogene conducted mRNA isolation and quality control (Bioanalyzer 2100 system, Agilent Technologies, Santa Clara, CA, USA) of the samples prior to mRNA seq and processing. RNA integrity was assessed using the RNA Integrity Number (RIN), with all samples meeting the quality threshold (RIN ≥ 7) to ensure reliable downstream analysis. Samples that were not deemed of sufficient quality were rejected. mRNA was purified from total RNA using poly-T oligo-attached magnetic beads. Following fragmentation, first-strand cDNA was synthesized using random hexamer primers, followed by second-strand cDNA synthesis.

Library preparation included end repair, A-tailing, adapter ligation, size selection, amplification, and purification. The kit used was the Novogene NGS RNA Library Prep Set (PT042). Libraries were quantified using Qubit and real-time PCR, and size distribution was assessed by Bioanalyzer. Raw FASTQ files underwent quality control using *fastp* v0.20.0 to remove adapter sequences, poly-N reads, and low-quality reads. Quality metrics (Q20, Q30, GC content) were calculated. In a further procedure, reads with >10% uncertain nucleotides, as well as > 50% low-quality nucleotides (Base Quality less than 5) per read, were removed. Thus, as raw sequencing reads often contain low-quality reads, they were filtered to clean the reads. Total raw reads per sample were 78,547 K–123,481 K, of which 88–92% passed filtering for the downstream analyses.

Clean reads were aligned to the human reference genome hg38; Ensembl, released December 2013, was employed, using HISAT2 v2.0.5, and gene counts were quantified with featureCounts v1.5.0-p3. Fragments per kilobase of transcript sequence per millions (FPKM) of each gene were calculated based on the length of the gene and read count mapped to this gene. Expected number of FPKM base pairs sequenced considers both the effect of sequencing depth and gene length (for the reads count). The method is currently the most commonly used method for estimating gene expression levels. For more details, see [55,56].

Differential expression analysis was conducted using DESeq2 v1.20.0 in R v3.22.5, with a design matrix incorporating age, sex, and sequencing batch as covariates to control for confounding. DESeq2 provides statistical programs for determining differential expression in digital gene expression data using models based on negative binomial distribution. Low-expression genes (fewer than 10 counts in at least three samples) were filtered out. Outlier detection was performed using Cook’s distance within DESeq2 to identify technical artifacts. Samples were filtered separately and normalized to eliminate differences in sequencing depth between samples. Moreover, library size normalization was performed using DESeq2’s median-of-ratios method [57]. To control for false discovery rate (FDR), the Benjamini–Hochberg procedure was applied across all contrasts simultaneously to maintain global FDR control [58]. Statistical significance was defined as adjusted *p*-value (padj) ≤ 0.05 and |log2 fold change| ≥ 1, with both up- and down-regulated genes reported separately for each contrast [58].

An initial assessment of clustering between the three was conducted using PCA (normalized counts and variance-stabilized transformed data), with variance explained by PC1 and PC2 reported. PCA plots were colored by group. Variance-stabilized transformed data were also used for sample-to-sample distance analysis. Further, uniquely expressed genes were defined by contrast-specific DEGs relative to threshold values, not to Venn diagram presence/absence [57]. FPKM cluster analysis was used for differential expression gene-clustering heatmap; the red color indicated genes with high expression levels, whereas the blue color indicated genes with low expression levels. Moreover, explicit contrasts were also examined. This means that the DEGs of each group were determined by their gene expression relative to a control group, i.e., set of samples from a relevant reference group.

To examine the characteristics of the chronic group versus the subclinical group and healthy control group, significant DEGs were analyzed using gene ontology (GO) and Kyoto Encyclopedia of Genes and Genomes (KEGG) pathway enrichment analyses. The analyses were performed with clusterProfiler v3.8.1 R package with gene-length bias correction. Significance was set at FDR < 0.05. Volcano plots included threshold lines for |log2 fold change| and adjusted *p*-values (enrichment results were directionality). The contrast between participants with only nsLBP (excluding those with co-occurring musculoskeletal pain) and the relevant control group was also examined. Batch effects were corrected for by matching mutual nearest neighbors, as previously described [59], to ensure reproducibility.

## 5. Conclusions

In summary, the present study suggests that patients with chronic nsLBP are different from individuals that have not developed chronic nsLBP, and it links such chronic pain to neuroplastic changes and/or low-grade inflammation. When comparing those with only chronic nsLBP to the subclinical group or the healthy controls, very clear differences regarding biological processes and pathways relating to neural activity, function, and wiring were uncovered. Thus, early recognition of the transition from subclinical to chronic nsLBP using the techniques described in this study may potentially pave the way for more accurate subgrouping or personalized therapeutic strategies targeting neuroplastic changes and inflammatory processes. From a clinical perspective, this offers hope, as it suggests that chronic pain can potentially be prevented if the process is halted early enough.

## Figures and Tables

**Table 1:** Participant characteristics.

	Control (n = 20)	Subclinical (n = 20)	Chronic (n = 19)
**Age, mean (SD)**	31.1 (8.4)	30.2 (8.4)	35.3 (10.0)
**Female, n (%)**	9 (45)	9 (45)	10 (53)
**Ethnicity, n (%)** European Māori Pacific People Asian Middle Eastern/Latin America/Africa Other Ethnicity	7 (35.0) - 2 (10.0) 7 (35.0) 3 (15.0) -	12 (60.0) - - 1 (5.0) 2 (10.0) 5 (25.0)	8 (42.1) - - 9 (47.4) - 2 (10.5)
**Education, n (%)** Primary School Secondary School Certificate/Diploma Higher Education (college/university < 4 years) Higher Education (college/university ≥ 4 years)	- 1 (5.0) 3 (15.0) 2 (10.0) 14 (70.0)	- 1 (5.0) 1 (5.0) 5 (25.0) 13 (65.0)	- 1 (5.3) 3 (15.8) 5 (26.3) 10 (52.6)
**Average pain intensity last 7 days** (NRS, 0–10), **mean (SD)**	-	2.2 (1.5)	4.4 (2.0)
**Currently experiencing pain elsewhere, n (%)** Yes No	2 (10.0) 18 (90.0)	9 (45.0) 11 (55.0)	9 (47.4) 10 (52.6)
**Duration of back pain, n (%)** 0–2 weeks 2–4 weeks 1–3 months 3–6 months 6–12 months >12 months		4 (28.6) 5 (35.7) - 2 (14.3) - 3 (21.4)	- - - - - 17 (89.4)
**Type of pain medication, n (%)** Over the counter (e.g., Paracetamol, Ibuprofen etc) Prescription (e.g., Tramadol) A combination of over the counter and prescription		3 (100.0) - -	5 (28.6) - 2 (28.6)
**Disability (RMDQ, range 0–24), mean (SD)**		3.0 (2.0)	4.9 (3.5)
**Kinesiophobia (FABQ-pa, range 0–24), mean (SD)**		4.8 (3.8)	10.9 (7.0)
**Pain catastrophising (PCS, range 0–52), mean (SD)**		6.6 (9.3)	15.5 (10.4)

FABQ-pa—Fear avoidance questionnaire-physical activity, n—number, NRS—numeric rating scale, PCS—Pain catastrophising scale, RMDQ—Roland Morris Disability Questionnaire, SD—standard deviation.

**Table 2:** Top four significant biological processes from gene ontology (GO) enrichment analysis comparing the chronic to the control group (top) or chronic to the subclinical group (bottom).

	Biological Process	Genes	p-adj.	Count
**Chronic vs. Control**	Axon guidance	*SPTBN4*, *NPHS1*, *CNTN2*, *RAP1GAP*, *KIRREL1*, *SEMA3B*, *EFNB3*, *EFNA2*, *SLIT3*, *NTN3*, *VAX1*, *RELN*, *SHANK3*, *PLXNB3*, *FGF8*, *EPHA8*, *SEMA3F*, *GBX1*, *GFRA1*, *SLIT2*, *NRXN3*, *GRB7*, *NFASC*, *NTRK1*, *UNC5C*, *BMP7*	0.00014	26
	Regulation of trans-synaptic signaling	*MAPK8IP2*, *GRM6*, *CNTN2*, *SLC6A9*, *CALB2*, *UNC13A*, *GRID1*, *GRID2IP*, *CA7*, *GRIN2D*, *ACHE*, *CHRNB4*, *SLC8A2*, *SYT12*, *ADRA1D*, *RELN*, *SYT7*, *CHRNB2*, *SHANK3*, *GRM4*, *ATP1A2*, *HAP1*, *GRIK3*, *CAMK2B*, *SHANK2*, *NTF4*, *NTRK1*, *SHISA7*, *CACNG5*, *SLC6A1*, *P2RX3*	0.00014	31
	Modulation of chemical synaptic transmission	*MAPK8IP2*, *GRM6*, *CNTN2*, *SLC6A9*, *CALB2*, *UNC13A*, *GRID1*, *GRID2IP*, *CA7*, *GRIN2D*, *ACHE*, *CHRNB4*, *SLC8A2*, *SYT12*, *ADRA1D*, *RELN*, *SYT7*, *CHRNB2*, *SHANK3*, *GRM4*, *ATP1A2*, *HAP1*, *GRIK3*, *CAMK2B*, *SHANK2*, *NTF4*, *NTRK1*, *SHISA7*, *CACNG5*, *SLC6A1*, *P2RX3*	0.00014	31
	Neuron projection guidance	*SPTBN4*, *NPHS1*, *CNTN2*, *RAP1GAP*, *KIRREL1*, *SEMA3B*, *EFNB3*, *EFNA2*, *SLIT3*, *NTN3*, *VAX1*, *RELN*, *SHANK3*, *PLXNB3*, *FGF8*, *EPHA8*, *SEMA3F*, *GBX1*, *GFRA1*, *SLIT2*, *NRXN3*, *GRB7*, *NFASC*, *NTRK1*, *UNC5C*, *BMP7*	0.00014	26
**Chronic vs. Subclinical**	Axon guidance	*BMP7*, *CNTN2*, *CYFIP1*, *EPHA8*, *FAM129B*, *FGF8*, *GBX1*, *GFRA1*, *KIRREL1*, *LGI1*, *LHX1*, *LHX3*, *NFASC*, *NPHS1*, *NTN3*, *PLXNB3*, *PLXND1*, *RAP1GAP*, *SEMA4A*, *SHANK3*, *SLIT2*, *SLIT3*, *SPTBN4*, *VAX1*	0.01242	24
	Adenylate cyclase-modulating G-protein coupled receptor signaling pathway	*APLP1*, *PTH1R*, *GRM6*, *GRIK3*, *CHGA*, *AVPR2*, *GLP2R*, *GNAT1*, *PTGER1*, *ADCY2*, *ADRA1D*, *GRM4*, *GPR176*, *GPR37L1*, *NOS1*, *GALR3*, *OPRD1*, *GALR2*, *GNA15*, *GPR78/FPR1*	0.01242	21
	Neuron projection guidance	*BMP7*, *CNTN2*, *CYFIP1*, *EPHA8*, *FAM129B*, *FGF8*, *GBX1*, *GFRA1*, *KIRREL1*, *LGI1*, *LHX1*, *LHX3*, *NFASC*, *NPHS1*, *NTN3*, *PLXNB3*, *PLXND1*, *RAP1GAP*, *SEMA4A*, *SHANK3*, *SLIT2*, *SLIT3*, *SPTBN4*, *VAX1*	0.01242	24
	G-protein coupled receptor signaling pathway, coupled to cyclic nucleotide second messenger	*APLP1*, *PTH1R*, *GRM6*, *GRIK3*, *CHGA*, *AVPR2*, *ANXA1*, *GLP2R*, *GNAT1*, *PTGER1*, *ADCY2*, *ADRA1D*, *GRM4*, *GPR176*, *GPR37L1*, *NOS1*, *GALR3*, *OR10H1*, *OPRD1*, *GALR2*, *GNA15*, *GPR78*, *FPR1*	0.01282	23

**Table 3:** Top significant pathway from Kyoto Encyclopedia of Genes and Genomes (KEGG) analysis comparing participants with only low back pain to control.

	Pathway	Genes	p-adj.	Count
**Chronic vs. Control**	Protein digestion and absorption	*COL20A1*, *SLC9A3P3*, *COL16A1*, *COL22A1*, *COL2A1*, *SLC8A2*, *ELN*, *ATP1A2*, *CPA1*, *SLC15A1*, *COL5A2*, *COL4A5*, *CTRB1*, *COL4A2*, *COL27A1*	7.5732 × 10^−6^	15
	Neuroactive ligand-receptor interaction	*GRM6*, *NPW*, *CHRNA2*, *UTS2R*, *F2*, *PTH1R*, *OPRD1*, *GRID1*, *AVPR2*, *GALR3*, *GRIN2D*, *GLP2R*, *CHRNB4*, *PTGER1*, *GNRH2*, *PYY*, *ADRA1D*, *CHRNB2*, *GRM4*, *C3P1*, *GALR2*, *GRIK3*, *RXFP4*, *KISS1R*, *CHRND*, *P2RX3*	1.0593 × 10^−5^	26
	Cholinergic synapse	*CREB3L3*, *ACHE*, *CHRNB4*, *CHRNB2*, *KCNJ4*, *KCNQ2*, *CACNA1A*, *GNG4*, *CAMK2B*, *CACNA1S*, *ADCY1*, *CACNA1B*	0.0012	12
	Glutamatergic synapse	*GRM6*, *GRIN2D*, *SLC1A6*, *PLA2G4F*, *SHANK3*, *GRM4*, *CACNA1A*, *GNG4*, *GRIK3*, *SHANK2*, *ADCY1*, *SLC38A3*	0.0013	12
	Axon guidance	*SEMA3B*, *EFNB3*, *FNA2*, *SLIT3*, *NTN3*, *NGEF*, *AC097065.1*, *PLXNB3*, *EPHA8*, *SEMA3F*, *SEMA6B*, *SLIT2*, *CAMK2B*, *UNC5C*, *RGMA*, *BMP7*	0.0031	16
**Chronic vs. Subclinical**	Neuroactive ligand-receptor interaction	*GPR156*, *PTH1R*, *SSTR3*, *GRID1*, *GRM6*, *F2*, *NPW*, *CHRNB4*, *GRIK3*, *AVPR2*, *GLP2R*, *PTAFR*, *PTGER1*, *C3P1*, *ADRA1D*, *GRM4*, *NPFFR1*, *GRIN2D*, *UTS2R*, *GALR3*, *OPRD1*, *CHRNA2*, *GALR2*, *LEP*, *FPR1*, *GAL*	0.0004	26
	Notch signaling pathway	*NOTCH3*, *NCOR2*, *RBPJL*, *CIR1*, *AC027279.1*, *AC105206.1*, *DLL3*, *DLL4*, *NOTCH1*, *HEY2*, *CATIP-AS2*	0.0140	11
	Protein digestion and absorption	*COL4A2*, *ELN*, *CPA1*, *ATP1A2*, *SLC9A3P3*, *COL20A1*, *SLC15A1*, *COL4A1*, *COL22A1*, *COL16A1*, *COL2A1*	0.0140	11
	ABC transporters	*ABCC6P1*, *ABCA11P*, *ABCD1P2*, *ABCC8*, *ABCA4*, *ABCG4*, *ABCB10P1*	0.0484	7

## Data Availability

The data utilized in this study are subject to restrictions, including legal and ethical considerations. Consequently, access to the data may be granted upon request to the corresponding author, contingent upon compliance with ethical guidelines, the General Data Protection Regulation (GDPR), and the relevant regulations governing patient rights in New Zealand and Norway.

## Supplementary Materials

The following supporting information can be downloaded at https://www.mdpi.com/article/10.3390/ijms27010287/s1.
