# Supplementary material for: Unique RNA Gene Expression Profile Is Seen in Chronic Non-Specific Low Back Pain
Source: Int J Mol Sci. 2025 Dec 27;27(1):287. doi: 10.3390/ijms27010287 (PMC12785320; doi:10.3390/ijms27010287)
Supplement: Supplementary file 1 [file ijms-27-00287-s001.zip › ijms-3990634-supplementary.pdf]

Supplementary tables and figures

**Supplementary Table S1.** Group comparison test for patient characteristics		
**Questionnaire**	**Test type**	**p-value**
PCS	T-test	p = 0.018*
FABQ- pa	T-test	p = 0.008*
RMDQ	T-test	p = 0.095*
NRS last 7 days	T-test	p = 0.002*
Ethnicity	Chi-square	p = 0.024
Education	Chi-square	p = 0.740
Duration of back pain	Chi-square	p = 0.000
Pain elsewhere	Chi-square	p = 0.021

*Only subclinical and chronic

FABQ-pa – Fear avoidance questionnaire - physical activity, **NRS** – numeric rating scale, **PCS** – Pain catastrophising scale, **RMDQ** – Roland Morris Disability Questionnaire

**Supplementary Table S2.** Overview of all uniquely expressed genes (gene names)		
**Control (n = 86)**	**Subclinical (n = 128)**	**Chronic (n = 139)**
AC005632.2	ABCB10	AC010343.1
AC005726.3	ABCE1	AC060780.3
AC009041.2	AC005831.1	AGPAT3
AC013264.1	AC055748.1	AL691432.1
AC016957.2	AC093227.1	ANXA2P2
AC025176.1	ACBD5	AP000759.1
AC103563.7	ADAM10	AP5B1
AC104794.2	AIDA	APBA3
AC245100.8	AL365273.1	ARRB1
ACTN1	ALG2	ATG2A
AL031727.1	ANKRD17	ATG3
AL136368.1	AP3M1	ATP6V0D1
AL844908.1	ARFGEF2	B3GALT6
AMY2B	ARID4B	BAHD1
ARFIP2	ATAD1	BID
BBS2	ATF1	BRI3
C1orf35	ATF2	BST1
CCDC61	ATP2A2	C15orf39
CERS5	BICRAL	C3AR1
COQ10A	BLOC1S6	C5AR1
COQ9	BTLA	C6orf226
DGCR8	C5orf24	CC2D1A
DHRS1	CASD1	CCR1
ENO3	CDK17	CCR5
EPHX2	CEBPZ	CD163
ERI3	CEMIP2	CD300C
FAM13A-AS1	CHMP2B	CD36
FAM153A	CPNE3	CD86
FBXO41	EIF1AXP1	CDA
FOLR3	ERMN	CHP1
GEMIN7	ETV6	CHST15
GMPPA	EXOC2	CPPED1
GPR155	FAM199X	CSF2RB
HAUS5	FAM208B	CTNNBL1
HSBP1L1	FAM3C	CYB5R3
HTRA2	FEM1B	DNPH1
IGHD3-16	FOXN2	DPYD
IGHD4-17	FUBP3	EIF4A3
ING5	GCC2	EMILIN2
ISYNA1	HBP1	EPSTI1
KCNK6	HIST2H2BE	EXOSC5
KCTD7	ICE1	FAM129B

KMT2E-AS1	ICOS	FBXO6
LAS1L	INTS8	FXR2
LRSAM1	KIAA1109	GFI1
MIR3677	KIAA2026	GHDC
MIR4507	KMT5B	GLB1
MRPS15	LIMS1	GM2A
MRPS2	LRPPRC	GTPBP1
MRPS6	LRRC8C	H2AFX
MTND1P23	MAN1A1	H6PD
MUTYH	MAP3K4	HGH1
NME8	MBP	HLA-DQA1
NPEPL1	MDN1	HPS6
ODF2	MGAT5	HSPA6
PAXIP1-AS1	MLLT10	HSPBP1
PHKG2	NCOA1	HTATIP2
PI4KAP1	NCOR1	IER3
PPP2R3C	NPAT	IFIT3
RBMX2	NR1D2	IGHD1-14
RF00561	NUP88	IGHD1-20
RP9	OXCT1	IGHD1-7
RPAIN	PCNX1	IGHD5-5
SLC17A9	PDXDC1	IGKV1-5
SLC35E2A	PHC3	IGLV1-40
SMPD4	PPIE	IGLV3-1
SMYD5	PPIL4	IGSF6
SNAPC4	PPP1R12A	ITPK1
SNORA26	PRDM4	JPT1
SNORD20	PRKAR2B	KCTD5
SNORD83A	PRKDC	KEAP1
SUGP1	PROSER1	KIAA0930
TARS2	PSMD10	LILRA2
TIMM9	PSME4	LINGO3
TMEM42	PTPN11	LRP1
TRIM52	PTPN4	MARCKS
TRMU	RAB5A	MED8
TTC19	RALGAPB	METTL13
WDR55	RF00586	MICB
WDR74	RN7SL834P	MIR6753
WDR91	RNU6-1016P	MPG
ZBTB25	RNU7-3P	MRPS12
ZC3H12D	SEC22B	MRPS7
ZFP62	SETDB2	MTCH2
ZNF581	SGPP1	NACC1
ZNF671	SLC25A36	NCOR2
	SLC25A46	NDRG1
	SLK	NHP2
	SMARCA2	NUTF2
	SMARCAD1	OAS3
	SNORD101	PEPD
	SPIN1	PIP5K1C
	SPTBN1	PLIN3
	SPTLC1	PLOD3
	SREK1	PLSCR1
	SSR1	PLXNA3
	STYX	POR
	TBL1XR1	PQLC1
	TGDS	PSMB2
	TIMM10B	PTAFR
	TLR10	PYM1
	TMED5	RAB31
	TMEM87B	RAB5IF
	TMF1	RETN
	TNFSF8	RF00003
	TNPO1	RF00019
	TRAJ14	RNASE2
	TRAJ19	RNU7-61P
	TRAJ21	RRBP1
	TRBV2	RRP7A

	TRBV6-5	RXRA
	TREML1	SEMA4B
	TRGJP1	SETD1A
	TWF1	SH3TC1
	TXLNG	SIGLEC1
	UBE2E3	SIRPA
	UGCG	SIRPB2
	WDR43	SLC16A3
	WWC3	SLC1A5
	XRN1	SLC4A2
	ZBTB44	SMCO4
	ZFAND1	SNHG7
	ZKSCAN8	SOCS3
	ZNF22	SQOR
	ZNF318	TBC1D10B
	ZNF518A	TBXAS1
	ZNF641	TLNRD1
	ZNF664	TMEM115
		TNRC18
		TRBV7-2
		TRIAP1
		UBALD1
		UBE2D1
		YIF1A
		ZC3H18
		ZC3H3
		ZNF414
		ZNF428
		ZNF865

Supplementary Figure S1. GO enrichment bar plot – control group

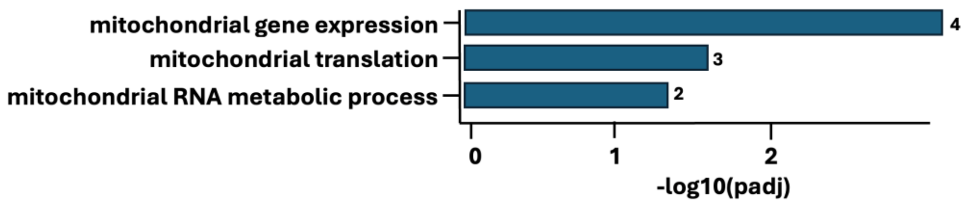

padj – p adjusted, **RNA** – ribonucleic acid, **RNase** - ribonuclease

Supplementary Table S3. Overview over statistically significant biological processes gene ontology (GO) functions in the control group.

Category	Count	High level GO category	padj
BP	4	mitochondrial gene expression	0.002
BP	3	mitochondrial translation	0.020
BP	2	mitochondrial RNA metabolic process	0.043

BP – biological process, **GO** – gene ontology, **padj** – p adjusted, **RNA** – ribonucleic acid

Supplementary Figure S2. Go enrichment dot plot subclinical group

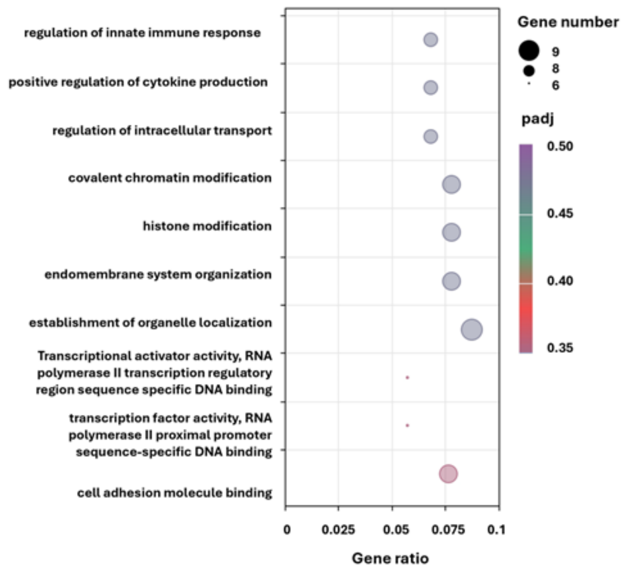

**Supplementary Table S4.** Overview over top five gene ontology pathways for the subclinical group			
**Category**	**Count**	**High level GO category**	**padj**
MF	5	protein N-terminus binding	0.146
MF	6	ligand-dependent nuclear receptor binding	0.367
MF	2	cell adhesion molecule binding	0.367
MF	3	cadherin binding	0.367
MF	2	syntaxin binding	0.367

GO – gene ontology, **MF** – molecular function

Supplementary Figure S3. Vulcano plot of differentially expressed genes (DEGs) comparing participants; subclinical group to the controls.

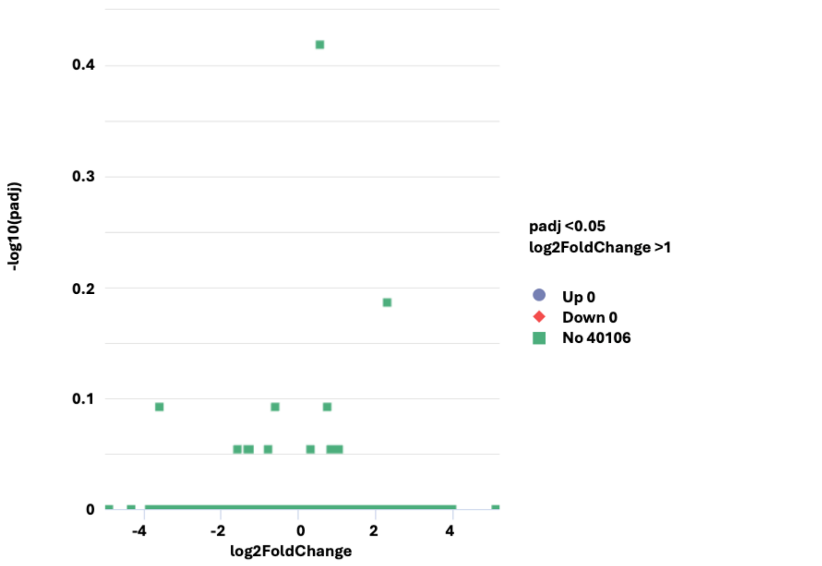

Supplementary Table S5. Top twenty significant pathway from KEGG analysis

Pathway	Genes	padj	Count
Protein digestion and absorption	COL20A1, SLC9A3P3, COL16A1, COL22A1, COL2A1, SLC8A2, ELN, ATP1A2, CPA1, SLC15A1, COL5A2, COL4A5, CTRB1, COL4A2, COL27A1	7.5732e-06	15
Neuroactive ligand-receptor interaction	GRM6, NPW, CHRNA2, UTS2R, F2, PTH1R, OPRD1, GRID1, AVPR2, GALR3, GRIN2D, GLP2R, CHRNB4, PTGER1, GNRH2, PYY, ADRA1D, CHRNB2, GRM4, C3P1, GALR2, GRIK3, RXFP4, KISS1R, CHRND, P2RX3	1.0593e-05	26
Cholinergic synapse	CREB3L3, ACHE, CHRNB4, CHRNB2, KCNJ4, KCNQ2, CACNA1A, GNG4, CAMK2B, CACNA1S, ADCY1, CACNA1B	0.0012	12
Glutamatergic synapse	GRM6, GRIN2D, SLC1A6, PLA2G4F, SHANK3, GRM4, CACNA1A, GNG4, GRIK3, SHANK2, ADCY1, SLC38A3	0.0013	12
Axon guidance	SEMA3B, EFNB3, FNA2, SLIT3, NTN3, NGEF, AC097065.1, PLXNB3, EPHA8, SEMA3F, SEMA6B, SLIT2, CAMK2B, UNC5C, RGMA, BMP7	0.0031	16
Calcium signaling pathway	RYR1, FGF22, GRIN2D, PTGER1, SLC8A2, ADRA1D, MYLK3, NOS2, MYLK2, FGF8, NOS1, CACNA1A, CAMK2B, CACNA1S, ADCY1, FGFR3, NTRK1, FGF18, CACNA1B, P2RX3	0.0077	20
ECM-receptor interaction	ITGB4, COMP, COL2A1, RELN, FREM2, COL4A5, LAMA1, COL4A2, LAMA3	0.0093	9
GABAergic synapse	HAP1, CACNA1A, GNG4, CACNA1S, ADCY1, SLC38A3, SLC6A12, SLC6A1, CACNA1B	0.0178	9
Insulin secretion	CREB3L3, ABCC8, KCNN1, ATP1A2, CAMK2B, CACNA1S, ADCY1, PCLO	0.0187	8
Regulation of actin cytoskeleton	APC2, ITGB4, F2, MYLPF, FGF22, AC097065.1, MYLK3, MYLK2, C7, FGF8, MYH14, MYL7, AC008567.1, FGFR3, AC116348.2, INSRR, BCAR1P1, FGF18, BCAR1	0.0187	19
Taste transduction	HTR3C, SCNN1G, ASIC2, GRM4, SCNN1B, CACNA1A, AC131235.2, P2RX3	0.0187	8

Phototransduction	GUCY2EP, CNGB1, GNAT1, GUCA1A, AC104581.3	0.0187	5
Adrenergic signaling in cardiomyocytes	CREB3L3, SLC8A2, ADRA1D, ATP1A2, PPP1R1A, CAMK2B, CACNA1S, ADCY1, TNNI3, SCN5A, CACNG5	0.0210	11
Cell adhesion molecules	CNTN2, CLDN3, CDH3, PTPRF, CLDN19, MAG, NRXN3, CDH5, HLA-DQB1, NFASC, AC116348.2	0.0215	11
Maturity onset diabetes of the young	PKLR, HNF1B, HNF4A, NKX6-1	0.0215	4
Apelin signaling pathway	NOTCH3, RYR1, SLC8A2, SERPINE1, MYLK3, NOS2, MYLK2, BORCS8-MEF2B, NOS1, GNG4, ADCY1, HNRNPA1P54	0.0215	12
ABC transporters	ABCC6P1, ABCG4, ABCC8, ABCB10P1, ABCA4, ABCB11	0.0267	6
Arrhythmogenic right ventricular cardiomyopathy	ITGB4, SLC8A2, ACTN3, NOS1, CACNA1S, AC008567.1, LAMA1, ACTN2, DES, CACNG5	0.0267	10
Taurine and hypotaurine metabolism	GGT6, GGT5, GGTLC2, GGT4P	0.0327	4
cGMP-PKG signaling pathway	CREB3L3, OPRD1, SLC8A2, ADRA1D, MYLK3, ATP1A2, CNGB1, MYLK2, BORCS8-MEF2B, CACNA1S, ADCY1, NPR1, HNRNPA1P54	0.0425	13
